# The impact of the implementation of the postpartum haemorrhage management guidelines at the first regional perinatal centre in Southern Kazakhstan

**DOI:** 10.1186/s12884-016-1027-4

**Published:** 2016-08-19

**Authors:** Ruta J. Nadisauskiene, Paulius Dobozinskas, Justina Kacerauskiene, Mindaugas Kliucinskas, Ismailov Zhumagali, Madina Kokenova, Jesengeldy Bekeshov, Saltanat Dzabagijeva, Aigul Sapargalijeva, Inna Glazebnaja, Gulmyra Konyrbajeva, Zijas Uteshova, Aina Tasbulatova

**Affiliations:** 1 Lithuanian University of Health Sciences, Eiveniu str. 2, 50167 Kaunas, Lithuania; 2 Health Department of the Southern Kazakhstan Region, Zeltoksan str. 20 “A”, Shymkent, 160012 South Kazakhstan Region Republic of Kazakhstan; 3 First Regional Perinatal Centre in Southern Kazakhstan, G. Iliaeva str. 142A, Enbekshinskii raion, Shymkent, 160011 South Kazakhstan Region Republic of Kazakhstan

**Keywords:** Postpartum haemorrhage, Management, Guidelines, Outcomes

## Abstract

**Background:**

Postpartum haemorrhage (PPH) remains one of the most common causes of maternal morbidity and mortality. Therefore, clearly written PPH management guidelines should be used in clinical practice. The aim of this study was to evaluate the effectiveness of the implementation of PPH management guidelines at the First Regional Perinatal Centre of Southern Kazakhstan (FRPC).

**Methods:**

Between 2012 and 2013 an interventional study was performed whereby the PPH management guidelines were implemented at the FRPC. All of the deliveries that were complicated by PPH 8 months before and 8 months after the intervention were analysed. Prevalence and severity of PPH, and the change in prevention, diagnostics and management of PPH was evaluated and statistical analysis using the SPSS 22.0 was performed.

**Results:**

There were in total 5404 and 5956 deliveries in the pre- and post-intervention periods, respectively. The rates of PPH and severe PPH decreased from 1.17 to 1.02 % (*p = 0.94*) and from 0.24 to 0.22 % (*p = 0.94*), respectively. Blood loss on average increased from 1055 to 1170 ml in the post-intervention period. The pharmacological treatment of postpartum haemorrhage with uterotonics was administered most frequently during both periods. After the implementation of the guidelines, the number of transfused units of packed red blood cells decreased from 4.76 to 2.48 units/case. In addition, the amount of transfused fresh frozen plasma decreased by 20 %. The number of conservative interventions and conservative operations increased from 7.9 to 52.7 % and from 3.9 to 48.6 %, respectively. The number of hysterectomies decreased from 23.7 % in pre-intervention to 8.1 % in the post-intervention period.

**Conclusions:**

The implementation of the PPH management guidelines had a positive effect on PPH prevention, diagnostics and management. It led to a more conservative aproach to the treatment of PPH. Therefore, clearly written PPH management guidelines, adapted for a particular hospital, should be developed and used in clinical practice.

## Background

Despite the efforts to reduce the rates of postpartum haemorrhage (PPH), it remains one of the most common causes of maternal morbidity and mortality [1]. Prior to the 1990s, a key strategy in reducing the PPH rate was training of individual health care providers. However, this strategy did not result in a reduction of PPH cases [2]. Therefore, attention was shifted towards team management of PPH. Consequently, various PPH management guidelines were developed at international and national levels. A systematic review of literature has shown that these guidelines can lower the PPH rate [3]. In addition, the best results are achieved when the guidelines are implemented during training courses and the whole team dealing with PPH attend them [3].

In this article we report the results of an international intervention conducted at the First Regional Perinatal Centre of the Southern Kazakhstan (FRPC). The PPH management guidelines were implemented at this tertiary hospital and the impact of the intervention on the number of PPH cases, and detection and management of PPH was then evaluated.

## Methods

### Study setting and design

**Fig. 1:**
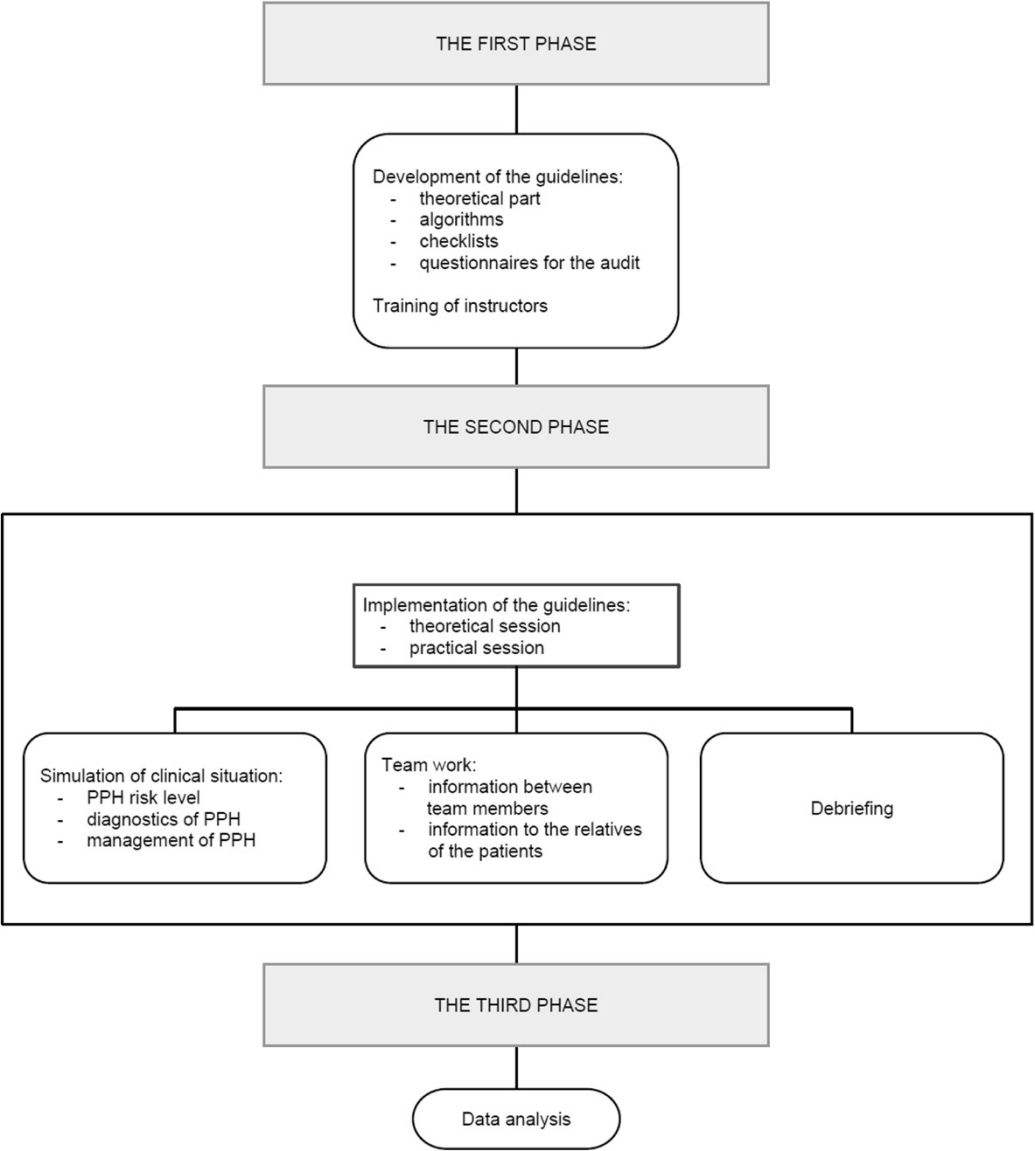
Implementation of the guidelines

An interventional study was developed as a result of collaboration between the Lithuanian University of Health Sciences (LUHS) and the Health Department of Southern Kazakhstan. The international project consisted of three phases (Fig. 1): preparation for the intervention, the intervention itself and the analysis of the results. The first and the third phases of the intervention were held at the Department of Obstetrics and Gynaecology of the LUHS, while the second phase was performed at the FRPC. The Department of Obstetrics and Gynaecology of the LUHS is a tertiary hospital which performs approximately 3200 deliveries annually. This accounts for 10 % of overall deliveries in Lithuania. The management of PPH is based on the national recommendations [4]. FRPC is the largest hospital in Southern Kazakhstan. This hospital accounts for 9100 births annually, or 11.6 % of overall deliveries in the region. Prior to this study, PPH cases were managed in accordance with the Order No 239 (07.04.2010) of the Ministry of Health of the Republic of Kazakhstan (available at: http://www.mzsr.gov.kz/) that included theoretical approach to a problem, and someone’s personal experience carrying out the procedures.

**Fig. 2:**
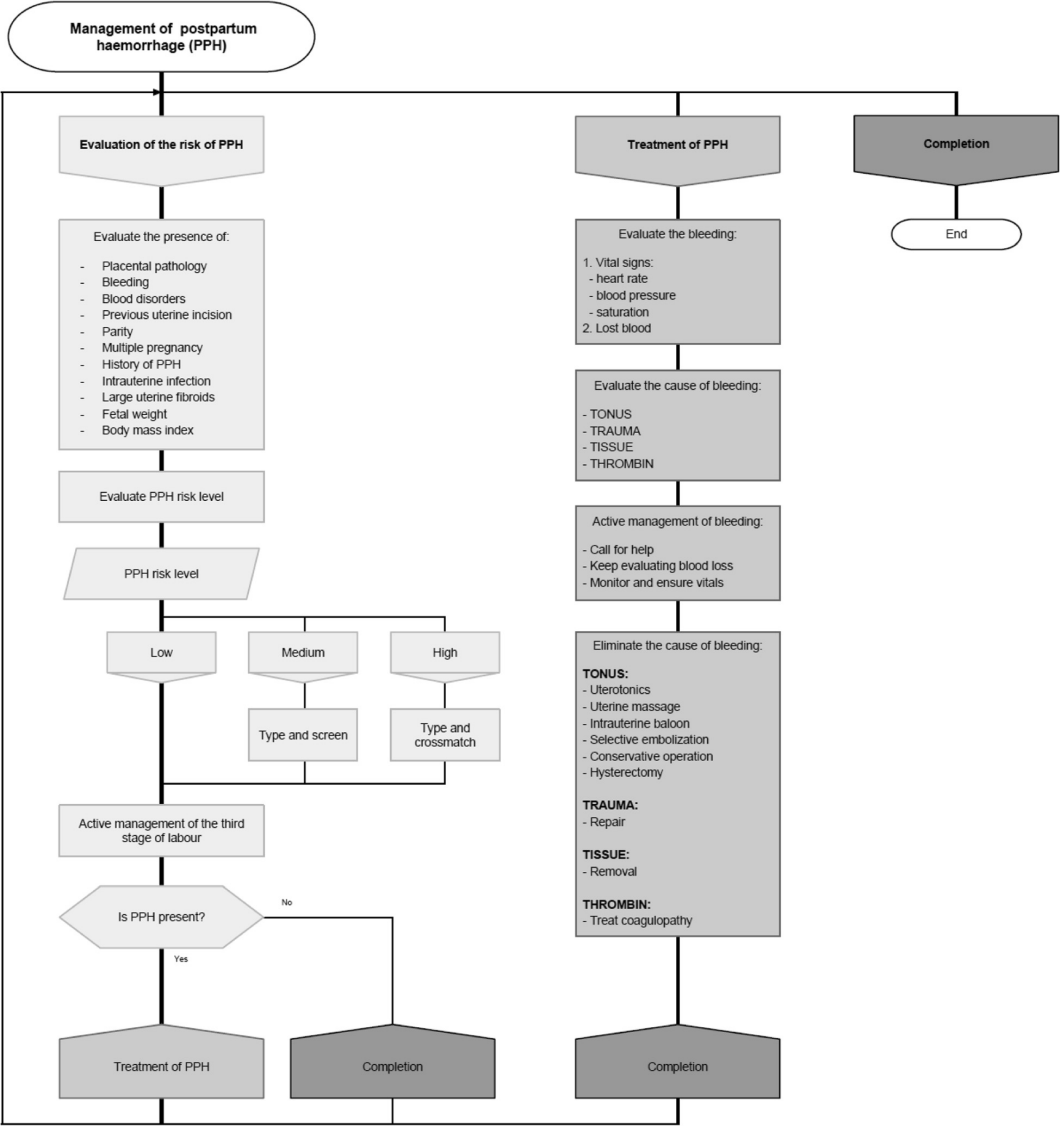
Management of postpartum haemorrhage

The first phase was initiated in April 2012. Unique guidelines were created for the FRPC. They were based on the recommendations for PPH management issued by the World Health Organization (WHO) [5], the International Federation of Gynaecology and Obstetrics (FIGO) [6] and the California Maternal Quality Care Collaborative (CMQCC) [7]. The guidelines consisted of a theoretical part, algorithms (Fig. 2), checklists and special questionnaires. Algorithms were created to facilitate the evaluation of the PPH risk, and to guide the medical practitioner in preventing, detecting and managing PPH. Checklists were designed to ensure proper documentation of blood loss, vital signs, medications and interventions. Special questionnaires were developed for the regular audit of PPH cases and the detection of weak points in management of PPH. All documents were developed by a group of specialists from Lithuanian and Kazakhstan. Preparation of the guidelines was preceded by discussions with the staff of the FRPC. Human resources, equipment and available finances were taken into account. The strengths and weaknesses of close collaboration among the obstetrics and anaesthesiology departments, the laboratory and the blood bank were also considered. After the guidelines were developed, they were reviewed by a group of leading Lithuanian and local specialists in obstetrics, midwifery, anaesthesiology and nursery. All these reviewers focused not only on the content of the guidelines but also on their adaptability to clinical practice. The observations made by the auxiliary staff were also taken into account. Some modifications were adopted. That was followed by the standardised training of four instructors (two obstetricians and gynaecologists, one anaesthesiologist and one midwife) who were responsible for implementing the guidelines at the FRPC. All of them are professionals in their field and have experience to lead courses, i.e. Advanced Life Support in Obstetrics (ALSO), etc.

After 6 months of the development of the guidelines and instructor training, the second phase was held at the FRPC. It lasted from October to November of 2012. This phase was dedicated to the implementation of the guidelines. This was performed in a course attended by PPH management-related personnel, including obstetricians and gynaecologists, midwives, anaesthesiologists, anaesthetists, nurses, representatives of the administration, as well as laboratory and auxiliary staff. Overall 70 trainees were trained. All of them got acquainted with the guidelines and went through a standardized course in PPH management in order to learn to use them in a proper way. The theoretical part was followed by practical sessions where trainees learned to determine and to minimize the PPH risk level, to properly instigate the active management of the third stage of labour, to accurately evaluate the blood loss from the beginning of the delivery by collecting the blood and weighing nursing materials, to respond to PPH cases in accordance with the guidelines, and to activate the checklist. The importance of informative registration of PPH cases together with proper audit of such cases was emphasised. The practical session was based on simulations of clinical cases and teamwork. This was organized by creating situations where activation of algorithms and checklists, teamwork approach, clearly defined responsibilities of team members, necessity of proper registration of events and used materials (medications, fluids, etc.) were practiced. Continuous sharing of information among team members and provision of information to the relatives of the patients were emphasized. After the simulation of a clinical case, debriefing was introduced as a crucial tool for the improvement of clinical practice.

The third phase consisted of data analysis. We used the data that had been recorded in the PPH audit checklists. We used these checklists for retrospective analysis of PPH cases that were registered before the intervention. We defined the period from September to December of 2012 as an intervention period. In September, all of the preparatory tasks were carried out. In October and November the course itself was held. December was defined as the transitional month. The 8 months before and 8 months after the intervention were defined as the pre- and post-intervention periods, respectively.

### Inclusion criteria

All of the deliveries that were complicated by PPH were included in the study. PPH was defined as a blood loss of ≥500 ml after vaginal delivery and ≥1000 ml after caesarean section. Severe PPH was defined as a blood loss of ≥1500 ml and/or unstable vital signs (heart rate, blood pressure and saturation) resulting from blood loss.

### Statistical analysis

A retrospective analysis of all of the deliveries in the pre- and post-intervention periods was performed. The data were obtained from case histories with the permission of the FRPC authorities. For data analysis we included all cases that fulfilled inclusion criteria. Data on PPH risk factors, mode of delivery, the cause of PPH, blood loss and PPH management were extracted. Comparison of obtained data from pre- *vs* post-intervention periods was performed. The chi-square test or Fisher’s exact test, as appropriate, was used for qualitative variables. For quantitative variables the unpaired *t*-test or exact Wilcoxon rank sum test, as appropriate, was used. The difference was statistically significant when *p* < 0.05.

## Results

There were in total 5404 and 5956 deliveries in the pre- and post-intervention periods, respectively. All the women in the post-intervention period were managed according to the implemented guidelines. The rates of PPH and severe PPH did not change in the post-intervention period (Table 1). The average blood loss at 1055 ml in pre-intervention period increased to 1170 ml in the post-intervention period. There were more multiparas and multiple pregnancies in the post-intervention period, but the prevalence of other PPH risk factors did not differ between the groups (Table 2). The number of women with anaemia decreased after the study. Active management of the third stage of labor, as the main tool for PPH prevention, stayed the same and was administered to all women in both groups.

**Table 1:** The postpartum haemorrhage rate

	Pre-intervention period (*n* = 5404)		Post-intervention period (*n* = 5956)		
	No.	%	No.	%	*p*
Overall postpartum haemorrhage	76	1.4	74	1.2	0.44
Postpartum haemorrhage	63	1.17	61	1.02	0.94
Severe postpartum haemorrhage	13	0.24	13	0.22	0.94

**Table 2:** Comparison of postpartum haemorrhage risk factors in the pre- and post-intervention periods

	Pre-intervention period (*n* = 76)		Post-intervention period (*n* = 74)		
	No.	%	No.	%	*p*
Obstetric factors					
Mean (SD) maternal age	29.86 (5.98)		31.28 (6.62)		
Anaemia	12	15.8	5	6.8	0.081
Multiparity	22	28.9	33	44.6	0.047
Previous lower segment caesarean section	7	9.2	10	13.5	0.406
Placental pathology	6	7.9	1	1.4	0.057
Multiple pregnancy	5	6.6	20	27	0.001
Induction of labour with oxytocin	9	11.8	15	20.3	0.159
Mode of delivery					
Spontaneous vaginal delivery	58	76.3	53	71.6	0.512
Lower segment caesarean section	18	23.7	21	28.4	0.512

The main cause of PPH was uterine atony. It was responsible for 88.2 and 89.2 % of PPH cases before and after intervention, respectively (Table 3).

**Table 3:** Etiology of postpartum haemorrhage

	Pre-intervention period (*n* = 76)		Post-intervention period (*n* = 74)		
	No.	%	No.	%	*p*
Uterine atony	67	88.2	66	89.2	0.842
Retained placenta	9	11.8	27	36.5	0.000
Lacerations of the birth canal	0	0	4	5.4	0.040

Among all of the PPH management options, uterotonics, including oxytocin, misoprostol and methylergonovine were administered most frequently in both periods (Table 4). Eight women in the pre- and three women in the post-intervention period did not get uterotonic as the first line management of PPH. Six women in the pre- and one woman in the post-intervention period had placental pathology and hysterectomies followed caesarean section. Two women in the pre-intervention period retained placenta, which had to be removed manually. Two women in the post-intervention period had lacerations of the birth canal that required repair. Following the training, the frequency of some conservative interventions (uterine massage and manual removal of placenta) for PPH management increased from 7.9 to 52.7 %. Other conservative interventions, such as intrauterine balloon tamponade, selective embolization, were not performed. After the implementation of the guidelines, the number of cases requiring blood transfusion increased (*p* = 0.018), even though the total number of transfused packed red blood cell units decreased from 4.76 to 2.48 units/case and the amount of transfused fresh frozen plasma decreased by 20 %. The content of operations changed as well. The number of conservative operations, that included B-Lynch suture, uterine artery or hypogastric ligation, increased from 3.9 to 48.6 % following the intervention. On the other hand, the number of radical operations, that included hysterectomies, decreased from 23.7 to 8.1 % in the post-intervention period (Table 4).

**Table 4:** Management of postpartum haemorrhage

	Pre-intervention period (*n* = 76)		Post-intervention period (*n* = 74)		
	No.	%	No.	%	*p*
Uterotonics	68	89.5	71	95.9	0.128
No. of cases when blood transfusion was performed	19	25	32	43.2	0.018
No. of transfused packed red blood cells/case	4.76		2.48		
No. of transfused plasma units	503		402		
Repair of lacerations	-	-	4	5.4	0.040
Manual removal of retained placenta	5	6.6	19	25.7	0.001
Uterine massage	1	1.3	20	27	0.000
Conservative operation	3	3.9	36	48.6	0.000
Radical operation	18	23.7	6	8.1	0.009

## Discussion

The most recent systematic review has revealed seven studies analysing the effect of successful implementation of the PPH management guidelines on clinical practice and their impact on the PPH rate [3]. The studies were conducted in the United States of America, several countries in Central and South America, Ireland, France, Spain and Pakistan. Herein, we report the effect of successful implementation of PPH management guidelines in Southern Kazakhstan.

In our study, the PPH and severe PPH rate decreased following the implementation of the guidelines. This is in line with the several previous studies, where the decrease in the PPH and severe PPH rates was reported after the implementation of the new PPH management guidelines [3]. The statistically insignificant decrease in our study may be explained by the fact that prior to the study, the blood loss evaluation was based on visual estimation and individual experience rather than on the objective measurements. During the intervention the staff were instructed to precisely evaluate the blood loss from the beginning of delivery. This was explained as an essential tool in detecting PPH early and managing it immediately. It is possible that as a result of that more PPH cases were detected in the post-intervention period than it would have been in the absence of the study. A similar conclusion was expressed by the other authors [8]. The intervention resulted in a precise quantification of blood loss and this may explain the slight increase in the total amount of blood lost in the post-intervention period.

The WHO’s Multicountry Survey on Maternal and Newborn Health defined the main risk factors for PPH [9]. During the intervention the trainees were taught to identify those factors and minimise their impact on PPH. Of all the proven risk factors for PPH, only the number of multiple pregnancies has increased in our study. But the number of the PPH and severe PPH has not changed. This might have been achieved by enhanced prevention, detection and management of PPH. The overall causes of the PPH did not change significantly between pre- and post-intervention periods. Similarly to our results, other researchers have demonstrated that the main cause of PPH was uterine atony [9]. In contrast to other studies, the number of retained placentas increased three fold following the intervention in our study. This might be explained by a better understanding of PPH etiology and more honest diagnostics after the intervention.

Similarly to the authors from California [10], we diagnosed PPH or severe PPH based not only on lost blood but on changes in vital signs as well. As reported in medical literature, this is an additional component to increase the sensitivity and specificity for detection of PPH [11].

Our study has shown a change in the management of PPH after the training. The PPH management became more conservative and more focused on the patient’s quality of life. Our study revealed that more PPH cases in the post-intervention period were initially managed using uterotonics. This has been reported by other authors as well [11–13]. Apart from that, roughly 12-fold increase in the number of conservative operations and uterine massages was determined. More uterine preserving operations after various interventions were reported by other authors as well [8, 10, 12, 14]. This may be a result of better skills and confidence achieved during the training. Unlike the number of conservative operations, the number of hysterectomies has decreased. This outcome corresponds to the findings by Shields et al. [10], but contradicts the results of studies performed in France, Pakistan or New York [8, 12, 15]. Some studies associate this increase with the growing caesarean section rate and its impact on future pregnancies [12, 15]. Although the number of women with a previous caesarean section was higher in the post-intervention period, this did not have any impact on the PPH or severe PPH rate or on the increase in the number of hysterectomies in our study.

Blood transfusions play a significant role in PPH management. Due to side effects, blood transfusions should not be performed too early. On the other hand, the delay can lead to irreversible changes. Although the total number of blood transfusions did not decrease after the intervention, the units of transfused packed red blood cells decreased nearly by half. This result correlates with Shields et al. findings [10]. The amount of transfused fresh frozen plasma also decreased. In addition to this, all the blood transfusions were performed through the peripheral, instead of the central venous catheter, which was common practice prior to the intervention. This suggests that only proper PPH management and a proper transfused component leads to a more successful and less aggressive intervention to deal with PPH.

The implementation of the PPH guidelines at the hospital of Southern Kazakhstan was carried out by a multidisciplinary team through theoretical and practical sessions. The purpose of sessions was not only to introduce the guidelines to the trainees, but also to teach them to use the guidelines in an appropriate way, to work in a team and to communicate with each other. This model was chosen because it is more effective than merely theoretical lectures. Studies show that practical training and simulations lead to a better knowledge and improvement of the skills, compared with conventional sessions [16, 17]. This may be achieved because of a better understanding of a situation from the perspective of all team members, improved communication among them and increased competence after solving a problem. Our study has also demonstrated a positive impact of practical introduction of the guidelines.

## Conclusions

The implementation of the PPH management guidelines had a positive impact on managing PPH. Both the guidelines and the way of implementation using practical sessions and simulations to use them have led to a better prevention, diagnostics and treatment of PPH. It also has led to a more conservative approach to the treatment of postpartum haemorrhage that has less negative effect on quality of life. Although many guidelines for PPH management exist, they should be adapted for each hospital with due regard to its specific capacities and limitations.

## Abbreviations

FRPC: The first regional perinatal centre of the Southern Kazakhstan
LUHS: Lithuanian University of Health Sciences
PPH: Postpartum haemorrhage
WHO: World Health Organization
